# Inspiration of the functional localization of a US naval hospital ship on a Chinese hospital ship

**DOI:** 10.1186/s40779-016-0076-3

**Published:** 2016-04-05

**Authors:** Da-Wei Li, Zhi-Cheng Zhang, Wen-Ya Zhu, Tao Sun

**Affiliations:** Department of Intensive Care Unit, Navy General Hospital, Beijing, 100048 China

**Keywords:** Medical support, International humanitarian assistance, Navy hospital ship

## Abstract

Through the experience of being stationed on the USS Mercy hospital ship (T-AH19) and a preliminary comprehension of the personnel and material arrangements, processing and functional formats, and the multi-platform contributions of US Navy hospital ships, we briefly introduce the characteristics of US hospital ships regarding medical support, emergency rescue exercises, communications and training, international humanitarian aid, etc. We discuss the function and responsibility of Chinese hospital ships, focusing on the investigation of the construction mode and positioning in the navy.

In July 2014, the Chinese Navy hospital ship Ark Peace was invited to participate in the 2014 Pacific Rim exercise (RIMPAC-2014), which is dominated by the US Navy. In the exercise, we were invited to visit the USS Mercy hospital ship (T-AH19). Over a 3-day visit, we gained a certain understanding of the treatment procedure, personnel arrangement and task functions of US Navy hospital ships, which caused us to consider the construction of our hospital ships.

The hospital ships of individual countries are used for medical support in wartime. For example, US Navy hospital ships were docked in the Persian Gulf for 56 days, treating more than 600 wounded people, including soldiers, prisoners and civilians, completing more than 500 operations, and playing a critical role in medical support for the war [1]. However, the operation of the hospital ship consumes large quantities of human and material resources. The maintenance cost of Mercy is 7 billion dollars per year. Therefore, in peacetime, the efficient operation of hospital ships is an important issue for all countries. In recent years, US hospital ships have undertaken several overseas medical support missions, such as Continuing Promise and Trans-Pacific Partnership, whereas our Ark Peace hospital ship undertook the Harmony Mission. In July, we participated in “Pacific Rim 2014”, which had a great impact on the world, contributing to improving the national image of China and displaying the Navy’s strength. These missions were all successful attempts at operating hospital ships without wars. In this article, we convey some of our experiences with the US*S* Mercy hospital ship and discuss the functions that Chinese Navy hospital ships should have in peacetime and the obligations that they should fulfill.

## Rapid reaction medical support platform

Providing medical support whenever necessary is the primary role of hospital ships. Currently, rapid response is a difficult problem that we have confronted with unexpected events. The USS Mercy hospital ship has a warehouse at a military harbor, where it keeps special medical equipment and medical supplies in reserve, ensuring the ability to respond to any emergency situation at all times. Regarding personnel arrangement, the US Navy has a specialized medical personnel database, and it can deploy personnel according to the scale and characteristics of a given missions. US Navy hospital ships depart with the necessary supplies as soon as a mission is acknowledged; it is not necessary that every person be present, which is a different aggregation strategy from ours. People who do not arrive at the ship at that time can take civil aircraft at specific times and locations. Based on this model, US Navy hospital ships can transition from guard state to readiness state within one week and can undertake medical support missions anywhere.

We allot some medical staff to the ship’s crew daily. At mission time, we must gather the necessary equipment and medical supplies temporarily according to concrete assignments, with personnel selected from each department temporarily. This model has the advantage of flexibility and can be adjusted at any time according to the nature of the mission. However, the disadvantages of this model are also obvious: it lacks a unified arrangement in personnel and materials, which can sometimes lead to wasted resources. Some mission units are far from the military harbor, and a great number of precise medical instruments and expensive medicines are inconvenient to gather, with a large amount of human resources, material and financial resources consumed [2]. Therefore, we suggest, as we learned from our experiences with the US Navy, that a specialized personnel database and warehouses for hospital ships be established, arranged by a specialized department, to ensure that hospital ships can provide medical support quickly with high quality whenever necessary.

## A normal naval medical exercise platform

Since it was commissioned, the Ark Peace hospital ship has participated in a number of domestic and overseas military exercises, including RIMPAC-2014, which was dominated by the US Navy, and all of them have gone well. Medical exercises are currently limited to missions, which mean that hospital ships arrange personnel and supplies temporarily with military exercises domestically and overseas, but there are no regular exercises without a mission. However, the US Navy hospital ships have regular medical exercises: they have routine medical rescue exercises each quarter of each year and overseas exercises each year [3]. The medical personnel databases of hospital ships have strict access systems; personnel in the databases must participate in a sufficient number of specialized exercises or they are deleted from the database and are disqualified from the medical missions of the hospital ship. While on the USS Mercy hospital ship, we had the great fortune to participate in a US Navy medical rescue exercise, and we were impressed with the degree of the crew’s specific standard operation processes and their strict evaluation system.

We should also make full use of the impressive platform of the hospital ship, insisting on medical exercises overseas as a routine practice, and the participants should include the personnel of the Navy’s health department and personnel in the medical databases. At the same time, we should improve the regular training course and establish complete systems for personnel training, admissions and evaluations to ensure that the hospital ship, as a base for naval medical exercises, and leading skilled medical personnel remain in a state of close cooperation for the naval medical services.

## Specialized naval medical communications and training platform

During Pacific Rim 2014, multilateral medical academic communications were conducted on the platforms of US and Chinese hospital ships up to 17 times on as many as 20 topics. Twelve of these communications were conducted on the USS Mercy hospital ship, 4 on the Peace Ark hospital ship, and 1 on the USS Peleliu (LHA-5). Through multilateral academic communications, we not only showed the other countries that participated in this exercise our medical support and rescue capabilities at sea, but we also gained useful experience from foreign navies and improved our academic and communication skills, which were rewarding experiences.

During our time on the USS Mercy hospital ship, we had the great fortune to experience Advanced Trauma Life Support (ATLS) training, which the personnel in the database of the USS Mercy hospital ship must attend at least once every five years. In addition to ATLS, medical staff must attend training for Advanced Cardiac Life Support (ACLS), Basic Life Support (BLS), etc. With a perfect simulation training system on the USS Mercy hospital ship, training personnel can simulate rescue, treatment and other types of operations on simulated humans; they can even simulate a real surgical operation.

The Peace Ark is the first hospital ship from our country, with incomparable advantages in terms of naval medical training; therefore, it should be used as a platform for naval medical communications and training. More academic communications should be undertaken between and within armies, both domestic and foreign, to increase the status of hospital ships in medical communications. At the same time, a complete simulation training system should also be built; on the one hand, it could be used to simulate training for medical personnel without a mission on the hospital ship, and on the other hand, it could also be used to administer medical training courses for the training and certification of naval medical personnel. Moreover, learning from the experiences of the USS Mercy hospital ship and communicating with local medical associations, we could provide training in trauma and emergency medicine nationwide. There are specialized military medical universities in our country and specialized naval medicine departments, but the content of our courses still lacks obvious naval characteristics. If the naval medicine bachelor’s training were combined with hospital ships and internships on hospital ships were mandatory, naval medical education would be greatly improved. All in all, as an extremely important force in naval medical support, hospital ships should not be ignored; with their academic status and the advantages of their platform, they should be fully used for naval medical service.

## Naval characteristics of military medical research platforms

As a large hospital on the sea, the hospital ship has a scale comparable to a third-grade class A hospital, with complete medical equipment and personnel, thus offering unique advantages to medical research. The US Navy conducts multi-faceted medical research with their hospital ships, attaining remarkable achievements in diving medicine, aviation medicine, naval medicine, etc. Since the Ark Peace hospital ship was commissioned, naval medicine personnel have been devoted to naval military medical research. However, for various reasons, they have been limited to epidemiological investigations, small-scale case analyses, etc., due to a lack of systemic prospective research projects. Therefore, we suggest that research centers be built in hospital ships in cooperation with interested researchers nationwide, to enable research on marine medicine and military medicine to promote the naval characteristics of military medical research.

## A more open humanitarian medical assistance platform

Humanitarian medical assistance is an important mission of US Navy hospital ships. Since 2005, US Navy hospital ships have been sent to countries in the developing world for international humanitarian medical assistance, including Continuing Promise and the Trans-Pacific Partnership, to show great power through military medical diplomacy. As platforms for humanitarian medical assistance, US Navy hospital ships have multiple personnel arrangements and vast financial resources. If the naval medical strength is insufficient to meet the demands of a mission, the US Navy receives support from the US Army, the US Air Force, US allies and the International Red Cross. In the case of Continuing Promise 2009, as a result of the United States’ wars in Iraq and Afghanistan, there was a shortage of naval medical personnel for this mission, so a medical team of 20 people from Canada, the Netherlands, and elsewhere and 130 volunteers dispatched by the International Red Cross participated in the mission. The medical service lasted for 4 months; it had a 26 million dollar budget and received a great deal of charitable donations and materials [4].

Since Harmony Mission 2010, the Ark Peace hospital ship has undertaken missions four times in 2010–2014, winning an outstanding political and diplomatic reputation. However, due to restrictions in personnel and material arrangements, mainly due to the Navy, we suggest that we could learn from the US Navy to establish a service pattern using the platform of the hospital ship, which the Navy would dominate, with other services and local medical departments and even nongovernment charities and foreign medical personnel participating as well. This pattern could not only solve the problem of funding and personnel shortages but also lead to more positive public opinion. During the recent Harmony Mission 2014 in Vanuatu, two Australian medical staff members who cooperated with Chinese medical staff, resulting in a successful medical service mission with foreign medical personnel. These types of communications will be increasingly frequent, followed by constant communication with foreign armies, thus influencing the promotion of hospital ships.

It has been nearly 30 years since the USS Mercy hospital ship entered service in the US Navy in 1986, but it has been only 7 years since Ark Peace entered active service in 2009. Therefore, abundant experience with the US Navy would be helpful to our hospital ships, including medical support, personnel arrangements, equipment support, etc. We believe that the precious resources of hospital ships will play more substantial roles in the near future and could be advantageous to medical support and to the medical development of the Chinese Army.

## Abbreviations

ACLS: Advanced cardiac life support
ATLS: Advanced trauma life support
BLS: Basic life support
